# Structured hashing with deep learning for modality, organ, and disease content sensitive medical image retrieval

**DOI:** 10.1038/s41598-025-93418-2

**Published:** 2025-03-14

**Authors:** Asim Manna, Dipayan Dewan, Debdoot Sheet

**Affiliations:** 1 Department of Artificial Intelligence, Indian Institute of Technology Kharagpur, Kharagpur, 721302 India; 2 Department of Electrical Engineering, Indian Institute of Technology Kharagpur, Kharagpur, 721302 India

**Keywords:** Image processing, Medical imaging, Machine learning

## Abstract

Evidence-based medicine is the preferred procedure among clinicians for treating patients. Content-based medical image retrieval (CBMIR) is widely used to extract evidence from a large archive of medical images. Developing effective CBMIR systems for clinical practice is essential due to the enormous volume of medical images of heterogeneous characteristics, viz. modalities, organs, and diseases. Deep neural hashing (DNH) has achieved outstanding performance and has become popular for fast retrieval on large-scale image datasets. However, DNH still needs to be improved for handling medical images, which often asks for knowledge of the semantic similarity of such characteristics. This work proposes a structure-based hashing technique termed MODHash to address this challenge. MODHash retrieves images with semantic similarity of the above characteristics as per user preference. The network of MODHash is trained by minimizing characteristic-specific classification loss and Cauchy cross-entropy loss across training samples. Experiments are performed on a radiology dataset derived from the publicly available datasets of Kaggle, Mendeley, and Figshare. MODHash achieves 12% higher mean average precision and 2% higher normalized discounted cumulative gain compared to state-of-the-art for top-100 retrieval. The characteristic-specific retrieval performance is evaluated, demonstrating that MODHash is an effective DNH method for evaluating user preferences.

## Introduction

X-rays, magnetic resonance (MR), computed tomography (CT), and ultrasound are the majorly used clinical imaging technologies that are driving the healthcare sector’s rapid digitalization^1^. They are utilized to capture different diseases from various organs. This resulted in the generation of a large archive of medical images. These imaging modalities play crucial roles in many cases, viz., medical diagnosis and treatment, providing valuable insights into patients’ internal structures and conditions^2^. This large amount of images can potentially transform treatment administration based on evidence, known as evidence-based medicine (EBM)^3,4^. EBM could be enhanced with the help of modern systems by giving importance to research evidence, clinical expertise, and patient values. Content-based medical image retrieval (CBMIR) is one example of such a system^5,6^.

The approach of CBMIR is to find the semantically closest neighbour from a collection of gallery images. However, the adoption of CBMIR systems in clinical practice could be improved by overcoming various challenges. These challenges include the operational efficiency of retrieval, the need for improved accuracy, reduction in the similarity gap, unavailability of pathological diagnosis associated with images, ineffective management of future data^7–9^.

**Fig. 1:**
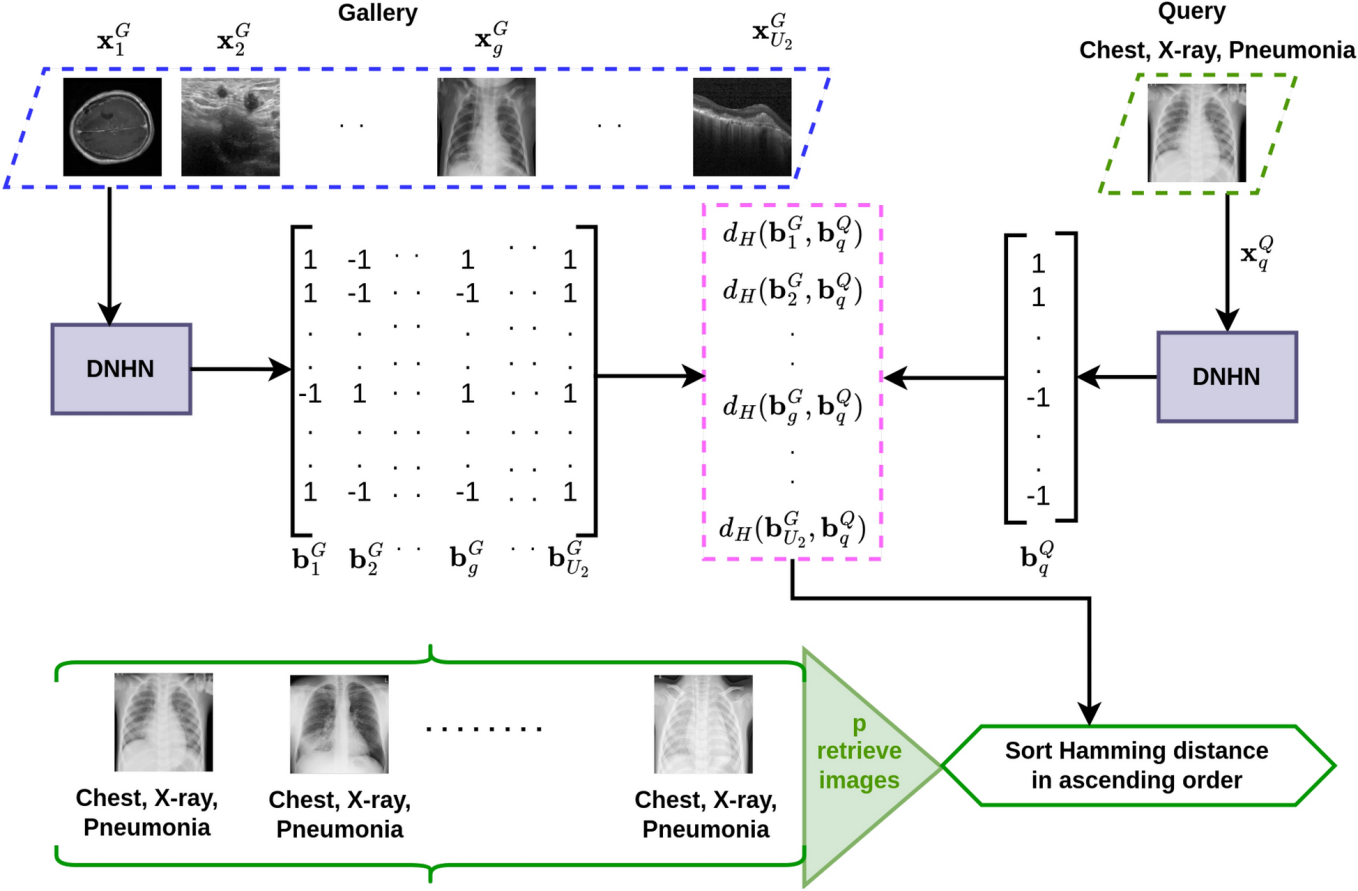
An overview of MODHash using deep neural hashing network (DNHN). $\textbf{x}_g^G \in \mathbb {R}^{1 \times M \times N}$ is an arbitrary image from gallery $(\textbf{X}_G)$ of medical images with different organ and pathologies, and $\textbf{x}_q^Q \in \mathbb {R}^{1 \times M \times N}$ is a query image with specific characteristics (modality- X-ray, organ- chest, pathology- pneumonia). $U_2$ represents the number of images present in the gallery. $\textbf{b}_g^G, \textbf{b}_q^Q \in \{-1, 1\}^{K \times 1}$ are hash codes of these images, respectively. Hamming distance $d_H(\textbf{b}_g^G, \textbf{b}_q^Q)$ is calculated to measure similarity with an image $\textbf{x}_g^G$. Then the top$-p$ $(p<<U_2)$ retrieved images $\{\textbf{x}_r^G, \textbf{x}_{r+1}^G, \dots , \textbf{x}_{r+p}^G\}$ are collected by sorting Hamming distances in ascending order, where $d_H(\textbf{b}_r^G, \textbf{b}_q^Q)$ is the minimum Hamming distance.

The CBMIR community often utilizes standard distance measurements and compact image features such as texture, color, and shape to find the closest matching images^5,10^. Comparing each query image and each sample in the archive is space and time-consuming^11^. It is not suitable for real-world scenarios with a large dataset. It has been shown that approximate nearest neighbor (ANN)^12^ methods may be a sufficient alternative in many applications that aim to perform large-scale information search and retrieval. The concept of deep neural hashing (DNH)^13,14^ has been introduced to retrieve images at high speed and lower storage demands compared to other retrieval methods. DNH is employed to transfer high-dimensional data to low-dimensional binary vectors with similarity preservation. The generated hash codes of images from DNH promote computationally efficient retrieval of images and is a widely studied solution for the problem of ANN search.

Image-based diagnosis is sensitive to characteristics featuring modality(s), organ(s), and disease(s). Improving EBM in order to prioritize based on certain of the above-mentioned characteristics requires the CBMIR system to be capable of characteristics-specific retrieval. It is generally well known that an organ in the human body can be affected by multiple diseases. Also, an organ can be investigated using multiple imaging modalities. Searching across different combinations of these variability becomes a challenging search problem. You et al. proposed a novel class-aware transformer module to enhance the learning of discriminative object regions with semantic structures^15^. Additionally, they introduced a principled approach to leverage the dataset for discovering similar yet distinct samples from different anatomical views, while employing a content discriminator and cross-cycle consistency loss to separate domain-invariant shared information from domain-specific features^16,17^.

Figiure 1 illustrates the overview of our image retrieval approach using DNH. In the last few years, multiple pairwise DNH frameworks have been developed based on semantic similarities, such as histopathology siamese deep hashing (HSDH)^18^, hybrid dilated convolution spatial attention (HDCSA)^19^, OrthoHash^20^, PCDH^21^, deep Cauchy hashing (DCH)^22^, HashNet^23^, etc. These previously developed DNH are not properly suitable for finding the similarity of modality, organs, and associated diseases because they only focus on semantic similarity^24^ between one characteristic of an image pair. By employing these approaches, it is possible to concentrate on either modality, organ, or disease, but not all three simultaneously. This work focuses on two objectives:

1. How can images be retrieved from a medical archive that consists of different combinations of such characteristics?
2. How can images be retrieved more accurately that are semantically similar to given specific characteristics?

The previous methods are applicable when all possible combinations of modalities, organs, and diseases are considered equal to different classes. In this case, characteristic-specific retrieval is not an option. As an example, the goal might be to retrieve images of the chest from any modality affected by pneumonia, images of chest CT affected by pneumonia, or images of any organ and modality affected by a specific disease. Previous methods fail in such cases. Hence, an effective mechanism to retrieve medical images based on the semantic similarity of such characteristics needs to be developed to help clinicians search through these large datasets. This paper proposes a structure-based CBMIR system called MODHash to address these challenges. Previous methods produce one hash code for one image at a time, which is expected to contain all information about different characteristics. But MODHash generates user preference hash code based on characteristics. The images that share multiple characteristics are retrieved through this structural property of MODHash.

The contributions to this work are as follows:

- To the best of our knowledge, this is the first attempt to retrieve images by considering the semantic similarity of modality, organs, and their associated diseases.
- An effective method is proposed for the CBMIR system, which produces a structure hash code with variable lengths for different characteristics.
- The popularly used metrics, including mean average precision (mAP) and normalized discounted gain (nDCG), are redefined with similarity scores in order to evaluate the proposed method.

## Related work

There are few traditional hashing methods for image retrieval without deep neural networks (DNNs)^25^, such as average hashing^26^, perceptual hashing^27^, locality sensitive hashing (LSH)^28^ etc. Traditional hashing techniques do not follow data distribution of a dataset, which is useless for large datasets in practical applications. Meanwhile, the generated code from images becomes image-independent hash code. However, lengthy codes are required to achieve high recall precision because the data distribution is not taken into consideration, which is useless for large datasets in practical applications. Learning-based hashing algorithms emerge as a workable solution for such retrieval systems by encoding images as compact hash codes with similarity preservation in the Hamming space^22^.

Several studies have previously concentrated on supervised hashing-based image retrieval. In 2011, Krizhevsky et al.^29^ mapped the images to hash codes for CBIR by using a deep autoencoder. A framework to learn compact similarity-preserving hash code for images called deep supervised hashing (DSH)^30^ has been proposed for image retrieval. Kang et. al.^31^ developed a DNN model for multi-view hashing. Semi-supervised hashing^32^ is mixed with a small amount of labeled data with a big amount of unlabeled data.

Preserving data similarity in the Hamming space is the primary objective of the majority of learning-based data-dependent hashing techniques^11^. Recent studies have extensively used deep learning, particularly convolutional neural networks (CNNs)^33^, to tackle the CBIR problem in medical images. However, the CBMIR task has yet to receive equal attention despite the complexity introduced by different image characteristics. A Siamese network with a customized loss function is proposed to enable effective feature identification for medical image retrieval^34^. In this work^35,36^, the authors have introduced techniques for multi-label image retrieval for large image sets. Dermoscopic image retrieval based on deep semantic hashing for clinical images of pigmented skin conditions has been proposed^19^. DenseHashNet extracts features from the original images and then introduces the spatial pyramid pooling (SPP) layer so that features at different scales can be extracted and multi-scale features fused with information from multiple regions^37^. Triplet-based deep hashing networks have been introduced to enhance the accuracy of ranking in medical image retrieval systems^38^. Attention-based triplet hashing (ATH) network is an end-to-end system that is designed to learn low-dimensional hash codes that preserve the categorization, ROI, and small-sample information^39^. A scalable hashing framework using energy-based modeling (SH-EBM) has been proposed for multi-label image retrieval^40^. Semantic similarity-based hashing approach viz. HashNet^23^, DCH^22^, and OrthoHash^20^ are very effective in projecting similar images into closer hamming space. HSDH is tailored for histopathology image retrieval, employing two shared-weight deep hashing models to produce hash codes^18^.

The loss functions of these approaches are generally focused on minimizing the loss of semantic similarity based on only one characteristic of a pair of images viz. either modality or organ or disease. These existing methods can be used to retrieve single or multi-label medical images from the same organ. This work extends these concepts to address the problem of CBMIR by learning similarity that is not restricted to any specific characteristic. Since DCH^22^ directly learns the Hamming distance from an image pair, their loss function is incorporated into our approach. In contrast, our work aims to extend the scope of CBMIR beyond specific multi-label characteristics to encompass multiple modalities, organs, and diseases. A structure-based DNH technique called MODHash is introduced to address this more comprehensive problem.

## Methodology

### Problem formulation

Consider a training set $\textbf{X}_{\mathfrak {T}} = \{\textbf{x}_{1}^{\mathfrak {T}}, \textbf{x}_2^{\mathfrak {T}}, \dots , \textbf{x}_i^{\mathfrak {T}}, \dots , \textbf{x}_{U_1}^{\mathfrak {T}} \}$. Let, $\textbf{y}_i^{\mathfrak {T}}= [\textbf{y}_{1i}^{\mathfrak {T}}, \textbf{y}_{2i}^{\mathfrak {T}}, \textbf{y}_{3i}^{\mathfrak {T}}]$ represents the label of image $\textbf{x}_i^{\mathfrak {T}} \in \mathbb {R} ^{1 \times M \times N}$. $\textbf{y}_{1i}^{\mathfrak {T}} \in \{0,1\}^{\lambda \times 1}, \textbf{y}_{2i}^{\mathfrak {T}} \in \{0,1\}^{\mu \times 1},$ and $\textbf{y}_{3i}^{\mathfrak {T}} \in \{0,1\}^{\nu \times 1}$ are the respective labels (one hot vector) of modality, organ and disease corresponding the image $\textbf{x}_i^{\mathfrak {T}}$. $\lambda$, $\mu$, and $\nu$ are the number of modalities, organs and diseases in the training set respectively. The semantic similarity $s_{ij}$ of an image pair $\textbf{x}_i^{\mathfrak {T}}$ and $\textbf{x}_j^{\mathfrak {T}}$ is defined as,

1 $$\begin{aligned} s_{ij} = {\left\{ \begin{array}{ll} 1, & \text {if }~~ \textbf{y}_i^{\mathfrak {T}} = \textbf{y}_j^{\mathfrak {T}} \\ 0, & \text {otherwise} \end{array}\right. } \end{aligned}$$

Here, a non-linear hash function $F:\mathbb {R}^{1 \times M \times N} \mapsto \{-1,1\}^{K \times 1}$ is pursued such that each image $\textbf{x}_{i}^{\mathfrak {T}} \in \mathbb {R} ^{1 \times M \times N}$ can be hashed and represented into a *K*-length binary hash code $\textbf{b}_i^{\mathfrak {T}} \in \{-1, 1\}^{K \times 1}$. The hash code $\textbf{b}_i^{\mathfrak {T}}$ is formed by concatenating horizontally three sub-hash codes $\textbf{b}_{1i}^{\mathfrak {T}} \in \{-1,1\}^{K_1 \times 1}, \textbf{b}_{2i}^{\mathfrak {T}} \in \{-1,1\}^{K_2 \times 1}, \textbf{b}_{3i}^{\mathfrak {T}} \in \{-1,1\}^{K_3 \times 1}$ respectively by corresponding to the characteristics of modality, organ, and disease. Mathematically, it can be written as,

2 $$\begin{aligned} F(\textbf{x}_{i}^{\mathfrak {T}})= \textbf{b}_i^{\mathfrak {T}} = [\textbf{b}_{1i}^{\mathfrak {T}}, \textbf{b}_{2i}^{\mathfrak {T}},\textbf{b}_{3i}^{\mathfrak {T}}] \end{aligned}$$

where $[\textbf{b}_{1i}^{\mathfrak {T}}, \textbf{b}_{2i}^{\mathfrak {T}},\textbf{b}_{3i}^{\mathfrak {T}}] \in \{-1, 1\}^{(K_1 + K_2+ K_3) \times 1}$ represents horizontal concatenation of three binary vectors of $\textbf{b}_{1i}^{\mathfrak {T}}, \textbf{b}_{2i}^{\mathfrak {T}},\textbf{b}_{3i}^{\mathfrak {T}}$.

MODHash aims to learn $F(\cdot )$ on a image pair $\textbf{x}_{i}^{\mathfrak {T}}~ \text {and} ~ \textbf{x}_{j}^{\mathfrak {T}}$ in a supervised manner such that $d_H(\textbf{b}_{i}^{\mathfrak {T}}, \textbf{b}_{j}^{\mathfrak {T}}) \rightarrow 0,d_H(\textbf{b}_{1i}^{\mathfrak {T}}, \textbf{b}_{1j}^{\mathfrak {T}}) \rightarrow 0,d_H(\textbf{b}_{2i}^{\mathfrak {T}}, \textbf{b}_{2j}^{\mathfrak {T}}) \rightarrow 0, d_H(\textbf{b}_{3i}^{\mathfrak {T}}, \textbf{b}_{3j}^{\mathfrak {T}}) \rightarrow 0$ if and only if $s_{ij}=1$, where $d_H: \{-1,1\}^{K \times 1} \times \{-1,1\}^{K \times 1} \longrightarrow \mathbb {R}$ represents the Hamming distance between two hash vectors. The variables and symbols are presented in Table 1.

**Table 1:** Symbol & notation table.

Symbol	Notation
$\textbf{X}_{\mathfrak {T}}$, $\textbf{X}_G$, $\textbf{X}_Q$	Train set, Gallery set, Query set
$\textbf{U}_1$, $\textbf{U}_2$, $\textbf{U}_3$	#samples in $\textbf{X}_{\mathfrak {T}}, \textbf{X}_G, \textbf{X}_Q$
$\textbf{x}_i^{\mathfrak{T}}$	*i*-th train image from $\textbf{X}_{\mathfrak {T}}$
$\textbf{x}_g^G$	*g*-th gallery image from $\textbf{X}_G$
$\textbf{x}_q^Q$	*q*-th query image from $\textbf{X}_Q$
$\textbf{y}_{1i}^{\mathfrak {T}}, \textbf{y}_{2i}^{\mathfrak {T}}, \textbf{y}_{3i}^{\mathfrak {T}}$	Modality, organ, disease labels
$\lambda , \mu , \nu$	#modalities, #organs, #diseases
$\textbf{h}_i, \textbf{b}_i^{\mathfrak {T}}$	Continuous and binary hash codes of $\textbf{x}_i^{\mathfrak {T}}$
$\textbf{b}_g^{G}$	Binary hash code of $\textbf{x}_g^G$
$\textbf{b}_q^Q$	Binary hash code of $\textbf{x}_q^Q$
$\alpha , \gamma$	Scale hyperparameters
*K*	Hash code length
$d_H(\cdot )$	Hamming distance

### Training procedure

The method consists of the following steps. *First*, the characteristic classification loss is minimized to get distinct features discriminative of the characteristics. *Second*, the Cauchy cross-entropy loss is employed on hash code to preserve similarity by minimizing the Hamming distance between hash codes of similar characteristics images. The detailed procedure of these steps is discussed as follows.

Initially, a modified version of AlexNet^41^ is adapted as the backbone architecture to learn the parameters, which is denoted as $\texttt {Encoder}(\cdot )$. $\texttt {Encoder}(\cdot )$ takes an input image $\textbf{x}_{i}^{\mathfrak {T}}$ and produces a feature vector, denoted as $\textbf{f}_i \in \mathbb {R}^{1024 \times 1}$.

3 $$\begin{aligned} \textbf{f}_i = \texttt {Encoder}(\textbf{x}_{i}^{\mathfrak {T}}) \end{aligned}$$

4 $$\begin{aligned} \begin{aligned} \texttt {Encoder}(\cdot )&\mapsto \texttt {Conv2D: 64c11w4s2p} \rightarrow \texttt {ReLU} \rightarrow \texttt {MaxPool2D: 3w2s} \rightarrow \texttt {Conv2D: 192c5w0s2p} \rightarrow \texttt {ReLU} \\&\rightarrow \texttt {MaxPool2D: 3w2s} \rightarrow \texttt {Conv2D: 384c3w0s1p} \rightarrow \texttt {ReLU} \rightarrow \texttt {Conv2D: 256c3w0s1p} \rightarrow \texttt {ReLU} \\&\rightarrow \texttt {Conv2D: 256c3w0s1p} \rightarrow \texttt {MaxPool2D: 3w2s} \rightarrow \texttt {Flatten} \rightarrow \texttt {Linear: 1024} \end{aligned} \end{aligned}$$

$\texttt {Encoder}(\cdot )$ is derived from the initial part of AlexNet, where the last two Linear: 4096 layers are replaced with a single Linear: 1024 layer. The goal is to represent images using a very low-dimensional latent vector for hash code construction, a single linear layer with significantly fewer nodes than the original AlexNet is included. Additionally, three subsequent sub-networks (discussed in the next subsection) are employed to generate three sub-hash codes, each capturing distinct characteristics and requiring additional model parameters.

**Fig. 2:**
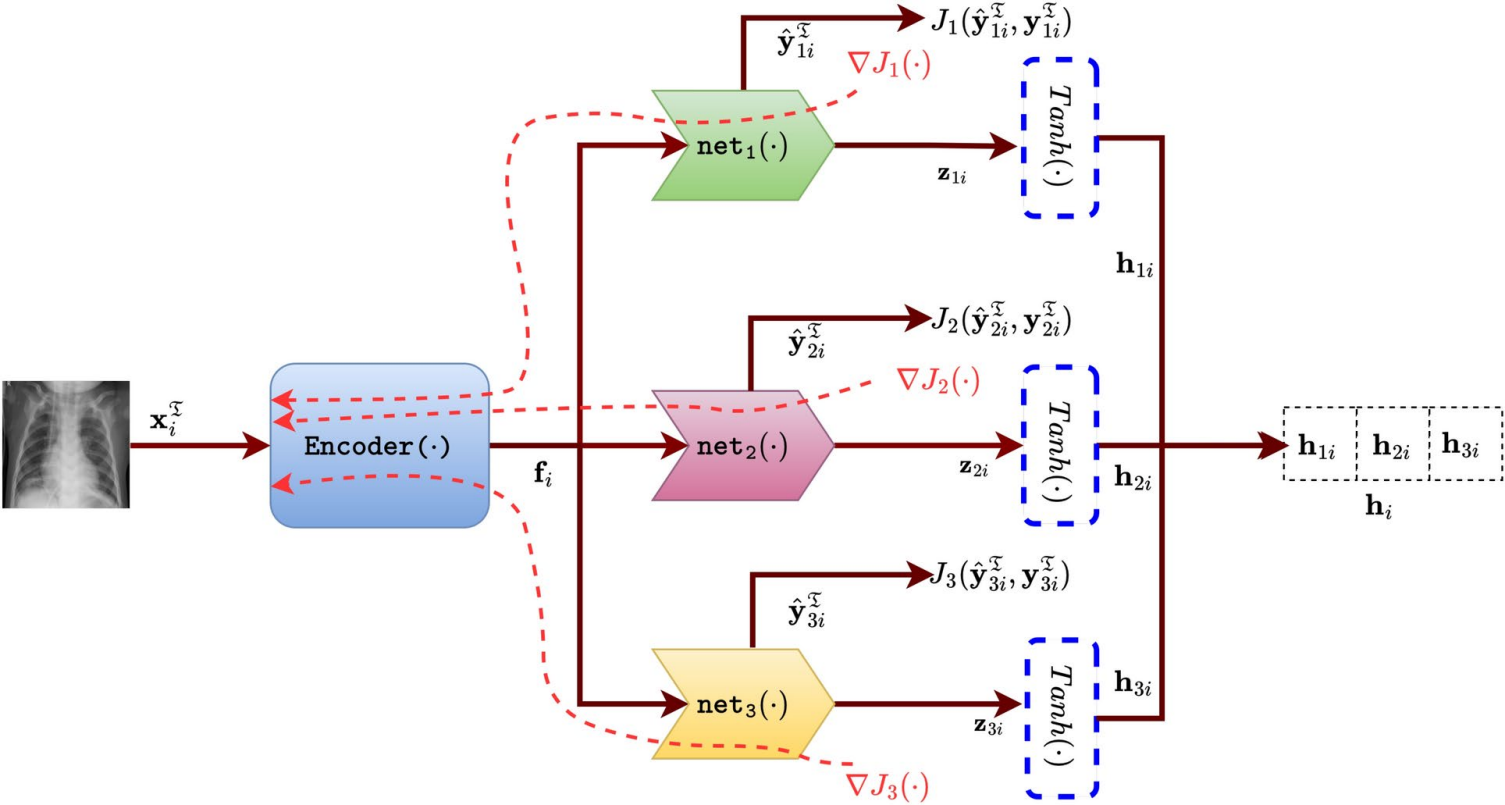
An overview of characteristic-specific classification loss and hash code generation. The input image $\textbf{x}_{i}^{\mathfrak {T}}$ passes through a CNN based network $\texttt{Encoder}(\cdot )$, and then followed by three sub networks $\mathtt {net_1}(\cdot ), \mathtt {net_2}(\cdot ),$ and $\mathtt {net_3}(\cdot )$. $\lambda , \mu , \nu$ denotes the number of modalities, organs, and diseases available in $\textbf{X}_{\mathfrak {T}}$. $\textbf{h}_i$ is the hash codes of $\textbf{x}_i^{\mathfrak {T}}$. The brown arrows represent the forward pass with the tensor, while the red dotted curves indicate the back-propagation of the characteristic-specific classification loss.

#### Characteristic-specific classification loss

Consider $\lambda$ number different modalities, $\mu$ number of different organs, and $\nu$ number of different diseases present in $\textbf{X}_{\mathfrak {T}}$. Three simple networks $\mathtt {net_1}(\cdot ), \mathtt {net_2}(\cdot ),$ and $\mathtt {net_3}(\cdot )$ are employed on $\textbf{f}_i$ for respectively classifying the othercharacteristics. $\hat{\textbf{y}}_{1i}^{\mathfrak {T}} \in \mathbb {R}^{\lambda \times 1}, \hat{\textbf{y}}_{2i}^{\mathfrak {T}} \in \mathbb {R}^{\mu \times 1}$, and $\hat{\textbf{y}}_{3i}^{\mathfrak {T}} \in \mathbb {R}^{\nu \times 1}$ are predicted characteristics and $\textbf{z}_{1i} \in \mathbb {R}^{K_1 \times 1}, \textbf{z}_{2i} \in \mathbb {R}^{K_2 \times 1}$, and $\textbf{z}_{3i} \in \mathbb {R}^{K_3 \times 1}$ are code features obtained from (5), (6), (7) respectively.

5 $$\begin{aligned} (\hat{\textbf{y}}_{1i}^{\mathfrak {T}}, \textbf{z}_{1i} )&= \mathtt {net_1}( \textbf{f}_i) \end{aligned}$$

6 $$\begin{aligned} (\hat{\textbf{y}}_{2i}^{\mathfrak {T}}, \textbf{z}_{2i} )&= \mathtt {net_2}( \textbf{f}_i) \end{aligned}$$

7 $$\begin{aligned} (\hat{\textbf{y}}_{3i}^{\mathfrak {T}}, \textbf{z}_{3i} )&= \mathtt {net_3}( \textbf{f}_i) \end{aligned}$$

8 $$\begin{aligned} \mathtt {net_1}(\cdot )&\mapsto \mathtt {Linear:512} \rightarrow (\mathtt {Linear:}\lambda , \texttt{Linear}:K_1) \end{aligned}$$

9 $$\begin{aligned} \mathtt {net_2}(\cdot )&\mapsto \mathtt {Linear:512} \rightarrow (\mathtt {Linear:}\mu , \texttt{Linear}:K_2) \end{aligned}$$

10 $$\begin{aligned} \mathtt {net_3}(\cdot )&\mapsto \mathtt {Linear:512} \rightarrow (\mathtt {Linear:}\nu , \texttt{Linear}:K_3) \end{aligned}$$

The above three networks generate two outputs, represented as a tuple. The first component of the tuple provides the characteristic-specific classification output, while the second component represents the characteristic-specific sub-hash code for an image. The hash code length is a user-defined, fixed value, while the number of classes depends on the dataset being used. Thus, the number of characteristic-specific classes and the hash code length are two independent variables. The motivation behind using these types of networks is to generate an accurate hash code representation based on the given information about the number of classes. All three characteristics are treated with equal importance in this work, and hence the identical architecture is used.

Given an input image $\textbf{x}_{i}^{\mathfrak {T}}$ and its corresponding class labels $\textbf{y}_{1i}^{\mathfrak {T}}, \textbf{y}_{2i}^{\mathfrak {T}}, ~ \text {and}~ \textbf{y}_{3i}^{\mathfrak {T}}$, the objective of this step is to minimize the classification losses $J_1(\hat{\textbf{y}}_{1i}^{\mathfrak {T}}, \textbf{y}_{1i}^{\mathfrak {T}}), J_2(\hat{\textbf{y}}_{2i}^{\mathfrak {T}}, \textbf{y}_{2i}^{\mathfrak {T}})$, and $J_3(\hat{\textbf{y}}_{3i}^{\mathfrak {T}}, \textbf{y}_{3i}^{\mathfrak {T}})$ for three respective characteristics. These losses are evaluated using cross-entropy (CE) loss. The total loss for this step is given by,

11 $$\begin{aligned} L_1 = \sum _{\textbf{x}_{i}^{\mathfrak {T}} \in \textbf{X}_{\mathfrak {T}}} \left[ J_1(\hat{\textbf{y}}_{1i}^{\mathfrak {T}}, \textbf{y}_{1i}^{\mathfrak {T}})+ J_2(\hat{\textbf{y}}_{2i}^{\mathfrak {T}}, \textbf{y}_{2i}^{\mathfrak {T}})+J_3(\hat{\textbf{y}}_{3i}^{\mathfrak {T}}, \textbf{y}_{3i}^{\mathfrak {T}}) \right] \end{aligned}$$

The above loss helps generate accurate characteristic predictions and, consequently, hash code generation from $\texttt {Encoder}(\cdot )$ and the three networks described.

#### Hash code generation

The $sign(\cdot )$ function is employed to convert the real valued hash to a binary hash representation. The hyperbolic tangent function $Tanh(\cdot )$ is used during training to avoid the vanishing gradient^22^ challenge faced on account of the $sign(\cdot )$ function. Thus, $\textbf{h}_i$ is utilized during training instead of $\textbf{b}_i^{\mathfrak {T}}$.

Three sub-hash codes $\textbf{b}_{1i}^{\mathfrak {T}} \in \{-1,1\}^{K_1 \times 1}, \textbf{b}_{2i}^{\mathfrak {T}} \in \{-1,1\}^{K_2 \times 1}, \textbf{b}_{3i}^{\mathfrak {T}} \in \{-1,1\}^{K_3 \times 1}$ of $\textbf{x}_{i}^{\mathfrak {T}}$ can be computed as,

12 $$\begin{aligned} \textbf{b}_{1i}^{\mathfrak {T}}&= sign(\textbf{h}_{1i}) =sign( Tanh(\textbf{z}_{1i}) \end{aligned}$$

13 $$\begin{aligned} \textbf{b}_{2i}^{\mathfrak {T}}&= sign(\textbf{h}_{2i}) =sign( Tanh(\textbf{z}_{2i})) \end{aligned}$$

14 $$\begin{aligned} \textbf{b}_{3i}^{\mathfrak {T}}&= sign(\textbf{h}_{3i}) =sign( Tanh(\textbf{z}_{3i})) \end{aligned}$$

The final hash code $\textbf{h}_i$ and $\textbf{b}_i^{\mathfrak {T}}$ can represented as,

15 $$\begin{aligned} \textbf{b}_i^{\mathfrak {T}}&= sign( \textbf{h}_i)\end{aligned}$$

16 $$\begin{aligned} \textbf{h}_i&= [\textbf{h}_{1i}, \textbf{h}_{2i},\textbf{h}_{3i}] \end{aligned}$$

The final real valued hash code $\textbf{h}_i \in [-1, 1]^{K \times 1} (K = K_1 + K_2 + K_3)$ of input image $\textbf{x}_i^{\mathfrak {T}}$ is concatenated by three sub-hash codes $\textbf{h}_{1i}^m \in [-1, 1]^{K_1 \times 1}$, $\textbf{h}_{2i} \in [-1, 1]$, $\textbf{h}_{3i} \in [-1, 1]^{K_3 \times 1}$. The training procedure of this step is illustrated in Fig. 2.

#### Cauchy cross-entropy loss

Learning the relationship between the manifested HD and semantic similarities is a crucial aspect of the retrieval task. Consider an image pair $\textbf{x}_{i}^{\mathfrak {T}}$ and $\textbf{x}_{j}^{\mathfrak {T}}$ and their hash codes $\textbf{h}_i$ and $\textbf{h}_j$ to be generated using (16). The Cauchy entropy loss is calculated between $s_{i,j}$ and $\hat{s}_{i,j}$ using binary cross-entropy, and is given by,

17 $$\begin{aligned} L_2&= -[s_{i, j} \log \left( \hat{s}_{i,j}\right) +(1-s_{i, j}) \log \left( 1-\hat{s}_{i,j}\right) ] \end{aligned}$$

18 $$\begin{aligned}&= s_{i, j} \log \left( \frac{d(\textbf{h}_i, \textbf{h}_j)}{\gamma } \right) + \log \left( 1 + \frac{\gamma }{d(\textbf{h}_i, \textbf{h}_j)} \right) \end{aligned}$$

where, predicted semantic similarity $\hat{s}_{i,j}$ is defined as,

19 $$\begin{aligned} \hat{s}_{i,j} = \frac{\gamma }{\gamma + d_H(\textbf{h}_i, \textbf{h}_j)} \end{aligned}$$

where, $\gamma$ is the scale parameter for Cauchy probability distribution^22^ and the Hamming distance between $\textbf{h}_i$ and $\textbf{h}_j$ is $d_H(\textbf{h}_i, \textbf{h}_j)$. The idea of (19) is that if predicted semantic similarities increase, the Hamming distance (HD) decreases, and vice versa.

#### Overall loss

The overall loss is computed as,

20 $$\begin{aligned} L = L_1 + L_2 + \alpha L_3 \end{aligned}$$

where, $\alpha$ is scale hyperparameter of the quantization loss ($L_3$) of a hash code $\textbf{h}_i$, which is defined by,

21 $$\begin{aligned} L_3 = \sum _{i} \Vert \textbf{h}_i - \textbf{b}_i^{\mathfrak {T}}\Vert _2 \end{aligned}$$

By minimizing *L*, thereby updating the parameters of $\texttt {Encoder}(\cdot )$, $\texttt {net}_\texttt {1}(\cdot )$, $\texttt {net}_\texttt {2}(\cdot )$ and $\texttt {net}_\texttt {3}(\cdot )$. The overall method is outlined in Algorithm 1.

**Algorithm 1:**
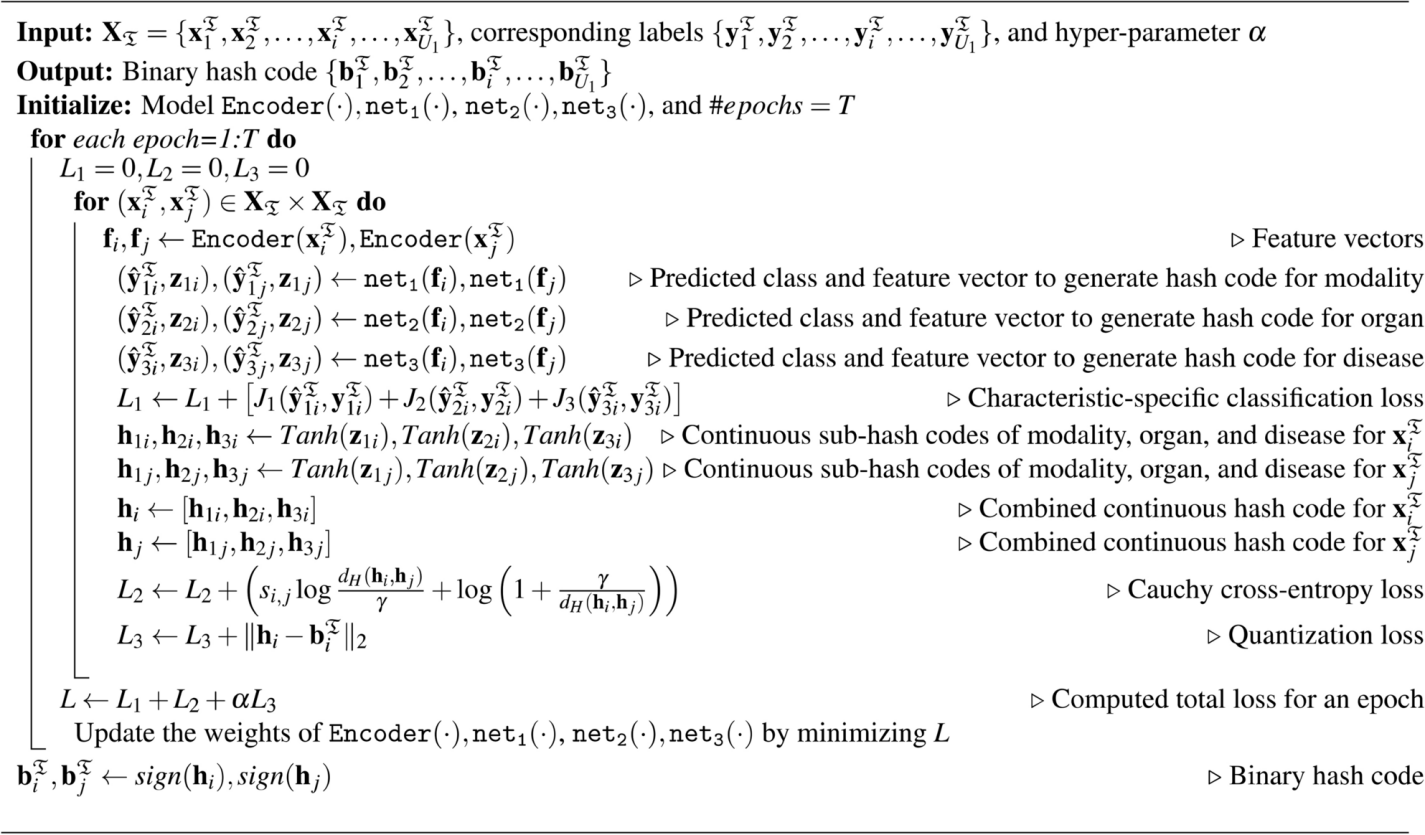
The pseudo code for the overall training pipeline of our method to learn and generate hash codes from images.

## Experiments

### Dataset

A number of publicly available datasets from different sources are combined together to construct a multi-organ, multi-modality, and multi-disease medical image dataset to validate the effectiveness of our proposed method. Details of these datasets are provided in Table 2. The combined dataset contains 13 different diseases with 5 modalities and 4 organs. All of these images are in grayscale format and resized to $224 \times 224$. The images in each dataset are split into three subsets: train $\left( \textbf{X}_{\mathfrak {T}} = \{\textbf{x}_1^{\mathfrak {T}}, \textbf{x}_2^{\mathfrak {T}}, \dots , \textbf{x}_{U_1}^{\mathfrak {T}}\} \in \mathbb {R}^{U_1 \times M \times N} \right)$, gallery $\left( \textbf{X}_{{G}} = \{\textbf{x}_1^{{G}}, \textbf{x}_2^{{G}}, \dots , \textbf{x}_{U_2}^{{G}}\} \in \mathbb {R}^{U_2 \times M \times N}\right)$, and query $\bigl (\textbf{X}_{{Q}} = \{\textbf{x}_1^{{Q}}, \textbf{x}_2^{{Q}}, \dots , \textbf{x}_{U_3}^{{Q}}\}$ $\in \mathbb {R}^{U_3 \times M \times N} \bigl )$. $U_1 = 22,935, U_2 = 7,164, U_3 = 1,700$. These groups are carefully created to ensure that there are no common samples between them. $\textbf{X}_{\mathfrak {T}}$ is used to train the network, while $\textbf{X}_{{G}}$ and $\textbf{X}_{{Q}}$ are used to assess performance of the trained model.

### Training settings

During the training process, the parameters of the networks are updated $\texttt {Encoder}(\cdot ), \texttt {net}_\texttt {1}(\cdot ), \texttt {net}_\texttt {2}(\cdot )$, and $\texttt {net}_\texttt {3}(\cdot )$. Adam optimizer is used to learn the parameters of the above four networks. The training continued with a batch size of 256 until losses and accuracy trends across epochs are saturated. The training has been initialized with a learning rate of 0.0001, and then the learning rate scheduler is used with patient 10 and factor 0.1. The value of hyperparameter $\alpha$ in (20) is assigned to 0.01. The experiments are conducted on a server equipped with 2x Intel Xeon 4110 CPUs, 12x8 GB DDR4 ECC Registered RAM, 2x4 TB HDD, 4x Nvidia GTX 1080Ti GPUs each with 11 GB DDR5 RAM, and running Ubuntu 20.04 LTS operating system. The algorithms are implemented using Python 3.7 with PyTorch 1.11 and Cuda 11.2.

**Table 2:** An overview of the dataset used in this work for experiment purposes.

**Organ**	**Modality**	**Disease**	**#Total images**
Brain	MRI [Link]	Glioma	3064
		Meningioma	
		Pituitary	
Chest	X-ray [Link]	Pneumonia	9900
		Covid	
		Normal	
	CT [Link]	Covid	8055
		Normal	
Breast	US [Link]	Benign	780
		Malignant	
		Normal	
Retina	OCT [Link]	CNV	10,000
		DME	
		DRUSEN	
		Normal	

This combined dataset contains four different organs, five different modalities, and 13 different diseases. There are total 15 different combinations of these characteristics.

### Structure hash code

Given that $\lambda$, $\mu$, and $\nu$ represent the number of classes for modality, organ, and disease, respectively, the hash code length should be greater than $\lceil \log _2(\lambda )\rceil$, $\lceil \log _2(\mu )\rceil$, and $\lceil \log _2(\nu )\rceil$ respectively. The hash code length is user-defined, subject to the constraints mentioned above. Selecting the optimal combination of $K_1$, $K_2$, and $K_3$ is entirely heuristic, as it depends on the data distribution and the number of nodes in the hash layer. The multiple models have been trained with different combinations of hash code length $K_1, K_2, K_3$. Initially, the values $K_1 = K_2 = K_3 = 8$ are chosen, resulting in a total hash code length of $K = K_1 + K_2 + K_3 = 24$. Given that there are five different modalities and four distinct organs, the same hash code length is maintained for both, i.e., $K_1 = K_2$. Since the number of total diseases is higher than the number of organs and modalities, $K_3$ remains equal or is increased in order to capture the necessary information adequately.

### Evaluation metrics

Let, $\textbf{b}_g^G$ is the binary hash code of an arbitrary image $\textbf{x}_{g}^{G} \in \textbf{X}_G$ and $|\textbf{X}_G| = U_2$. $d_H(\textbf{b}_g^G, \textbf{b}_q^Q)$ is the HD between $\textbf{b}_g^G$ and $\textbf{b}_q^Q$, where $\textbf{b}_q^Q$ is the hash code of a query image $\textbf{x}_{q}^{Q} \in \textbf{X}_Q$. The relationship between cosine similarity and normalized Euclidean distance between $\textbf{b}_g^G$ and $\textbf{b}_q^Q$ of length *K* can be expressed as follows:

22 $$\begin{aligned} \begin{aligned} d_H(\textbf{b}_g^G, \textbf{b}_q^Q) = \frac{K}{4} \left\Vert \frac{\textbf{b}_g^G}{\Vert \textbf{b}_g^G \Vert }_2 -\frac{\textbf{b}_q^Q}{\Vert \textbf{b}_q^Q \Vert }_2 \right\Vert ^{2}_{2}\\ \hspace{-0.1cm}= \frac{K}{2}\left( 1- cos(\textbf{b}_g^G, \textbf{b}_q^Q)\right) \end{aligned} \end{aligned}$$

Retrieval performance is evaluated using two metrics: mAP and nDCG^13^. The quality of retrieval is measured using mAP, while nDCG assesses the rank quality of the retrieved images across all query images. The computation process for both of these metrics is discussed in the following subsections.

#### Mean average precision

*mAP*@*p* represents the mAP for the top-*p* retrieved images from $\textbf{X}_G$. It is calculated by finding the average precision ($AP_q@p$) for each $\textbf{x}_{q}^{Q} \in \textbf{X}_Q$ based on the top-*p* retrieved images from $\textbf{X}_G$. Let, $\textbf{x}_r^{G}$ is the *r*-th ranked image from top-*p* retrieve images. Then $AP_q@p$ is defined as,

23 $$\begin{aligned} AP_q @p =\frac{\sum _{r=1}^{p} P_q(r) R^{mAP}_q(r)}{\sum _{r=1}^{p}R^{mAP}_r(r)} \end{aligned}$$

where, $P_q(r)$ denotes the precision for the top-*r* retrieval of query image $\textbf{x}_{q}^{Q}$ and is defined by,

24 $$\begin{aligned} P_q (r) = \frac{\sum _{r=1}^r R^{mAP}_q(r)}{r} \end{aligned}$$

25 $$\begin{aligned} R^{mAP}_q(r)= {\left\{ \begin{array}{ll} \ 1, & \text {if } ~~ \textbf{y}_r^G = \textbf{y}_q^Q\\ 0, & \text {otherwise } \end{array}\right. } \end{aligned}$$

Finally,

26 $$\begin{aligned} mAP@p = \frac{1}{U_3} \sum _{\textbf{x}_{q}^{Q} \in \textbf{X}_Q} AP_q @p \end{aligned}$$

**Table 3:** An overview of overall performance of our proposed method with different hash code lengths.

**Total Bits**	**Bit Structure**	**nDCG@10**	**nDCG@100**	**nDCG@1000**	**mAP@10**	**mAP@100**	**mAP@1000**
24	8+8+8	0.9704	0.9693	0.9759	0.8318	0.7966	0.7759
26	8+8+10	0.9686	0.9700	0.9756	0.8398	0.8028	0.7834
28	8+8+12	0.9728	0.9720	0.9774	0.8528	0.8077	0.7916
30	10+10+10	0.9666	0.9694	0.9757	0.8326	0.7916	0.7753
32	10+10+12	0.9664	0.9704	0.9762	0.8368	0.8030	0.7881
34	10+10+14	0.9706	0.9709	0.9769	0.8418	0.8025	0.7784
36	12+12+12	0.9703	0.9722	0.9771	0.8343	0.8070	0.7857
38	12+12+14	0.9701	0.9725	0.9775	0.8487	0.8083	0.7883
40	12+12+16	0.9686	0.9716	0.9773	0.8450	0.8082	0.7885
42	14+14+14	0.9679	0.9712	0.9776	0.8439	0.8075	0.7913
44	14+14+16	0.9658	0.9687	0.9763	0.8327	0.7994	0.7822
46	14+14+18	0.9653	0.9692	0.9759	0.8315	0.8005	0.7833
48	16+16+16	0.9690	0.9709	0.9766	0.8328	0.7979	0.7851
50	16+16+18	0.9648	0.9681	0.9763	0.8281	0.7920	0.7816
52	16+16+20	0.9669	0.9691	0.9753	0.8219	0.7971	0.7748
54	18+18+18	0.9659	0.9692	0.9757	0.8313	0.7945	0.7726
56	18+18+20	0.9714	0.9705	0.9773	0.8482	0.8039	0.7905
58	18+18+22	**0.9772**	**0.9748**	**0.9786**	**0.8554**	**0.8184**	**0.7965**
60	20+20+20	0.9582	0.9673	0.9760	0.8151	0.7946	0.7825
62	20+20+22	0.9636	0.9687	0.9760	0.8250	0.7966	0.7801
64	20+20+24	0.9692	0.9713	0.9777	0.8337	0.8037	0.7845

Bold values represent the best performance score among all the methods.

#### Normalized discounted cumulative gain

First, $DCG@p$ is calculated for the top-$p$ retrieved results, followed by the computation of $nDCG@p$ for top-$p$ retrieval. The mathematical formulation for $DCG_{q}@p$ of query image $\textbf{x}_{q}^{Q}$ is given by,

27 $$\begin{aligned} DCG_{q}@p = \sum _{r=1}^{p} \frac{2^{R^{nDCG}_q(r)}-1}{log_{2}(r+1)} \end{aligned}$$

This is normalized by dividing it by the maximally achievable value, known as the Ideal DCG (iDCG).. Finally to obtain,

28 $$\begin{aligned} nDCG_{q}@p = \frac{DCG_{q}@p}{iDCG_{q}@p} \end{aligned}$$

where $iDCG_{q}@p = DCG_{q}@p$ of ideal ranking or best possible ranking. The relevance score for image $\textbf{x}_{q}^{Q}$ is defined by,

29 $$\begin{aligned} R^{nDCG}_q(r)= V \end{aligned}$$

where $V \in \{0, 1, 2, 3\}$ represents the number of characteristics of $\textbf{x}_{q}^{Q}$ and $\textbf{x}_{r}^{G}$ is matched.

Finally,

30 $$\begin{aligned} nDCG@p = \frac{1}{U_3} \sum _{\textbf{x}_{q}^{Q} \in \textbf{X}_Q} nDCG_q @p \end{aligned}$$

## Results & discussion

### Overall performance

**Fig. 3:**
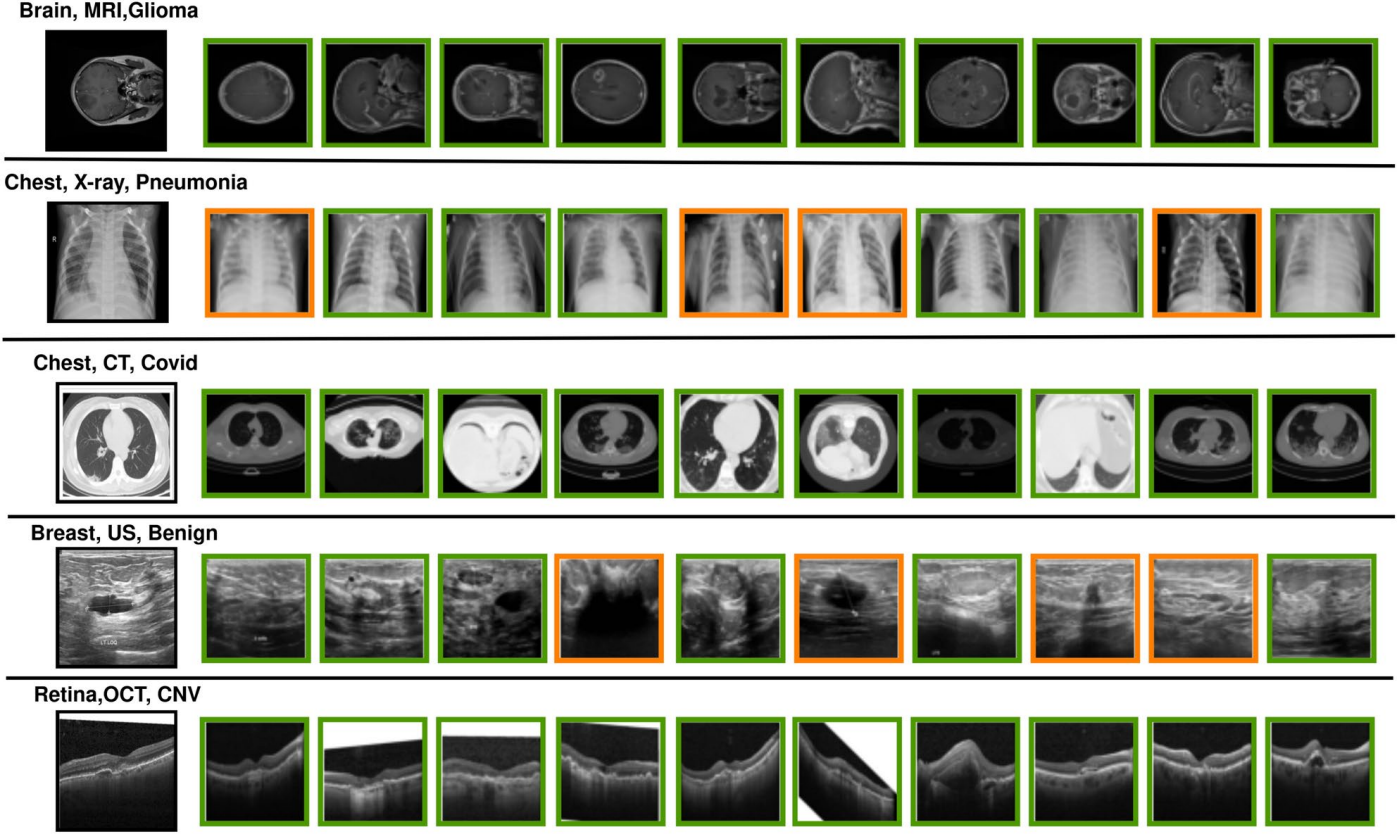
This figure illustrates the retrieved images from the gallery for a given query image. The leftmost column shows the query images along with their labels for modality, organ, and disease. The remaining columns display the top 10 retrieved images for each query. Retrieved images outlined in green boxes indicate matches across all three characteristics, while those in orange boxes represent instances where at least one characteristic (in this case, the disease) does not match.

Evaluation of 21 different variants of MODHash is conducted, each denoted as $MODHash-K$, where each variant corresponds to a distinct value of *K*. The performance assessment is carried out using quantitative metrics *mAP*@*p* and *nDCG*@*p* for $p=\{10, 100, 1000\}$. The summarized results are presented in Table 3. All of these variants exhibited very similar performance levels. However, $MODHash-58$ stands out with the most promising results, achieving *mAP*@10 score of 0.8336 and *nDCG*@10 score of 0.9692. In contrast, $MODHash-24$ performed less effectively. It is observed that a model performs well when it follows $K_{1} = K_{2} = K_{3}-4$. The top 10 retrieved images from $\textbf{X}_G$ are also attached in Fig. 3.

### Comparison with SOTA

Since our training process utilizes image pairs, the retrieval performance of our method is compared with that of pairwise deep hashing methods, such as HSDH^18^, HDCSA^19^, OrthoHash^20^, PCDH^21^, DCH^22^, HashNet^23^. The performance is compared with seven possible characteristic cases viz. $\mathcal {M}$ represents modality specific, $\mathcal {O}$ represents organ specific, $\mathcal {D}$ represents disease specific, $\mathcal{M}\mathcal{O}$ represents modality-organ specific, $\mathcal{M}\mathcal{D}$ represents modality-disease specific, $\mathcal{O}\mathcal{D}$ represents organ-disease specific, $\mathcal {MOD}$ represents modality-organ-disease specific. Tables 4 and 5 display the values of *mAP*@*p* and *nDCG*@*p*, respectively for each model. These metrics are redefined for each characteristic-specific experiment as follows:

- $R^{mAP}_q(r), R^{nDCG}_q(r) \in \{0, 1\}$ for single characteristic-specific i.e, $\mathcal {M}$, $\mathcal {O}$, and $\mathcal {D}$.
- $R^{mAP}_q(r) \in \{0, 1\}, R^{nDCG}_q(r) \in \{0, 1, 2\}$ for double characteristic-specific i.e, $\mathcal{M}\mathcal{O}$, $\mathcal{O}\mathcal{D}$, and $\mathcal{M}\mathcal{D}$.
- $R^{mAP}_q(r) \in \{0, 1\}, R^{nDCG}_q(r) \in \{0, 1, 2, 3\}$ for $\mathcal {MOD}$, as discussed in Table .

**Fig. 4:**
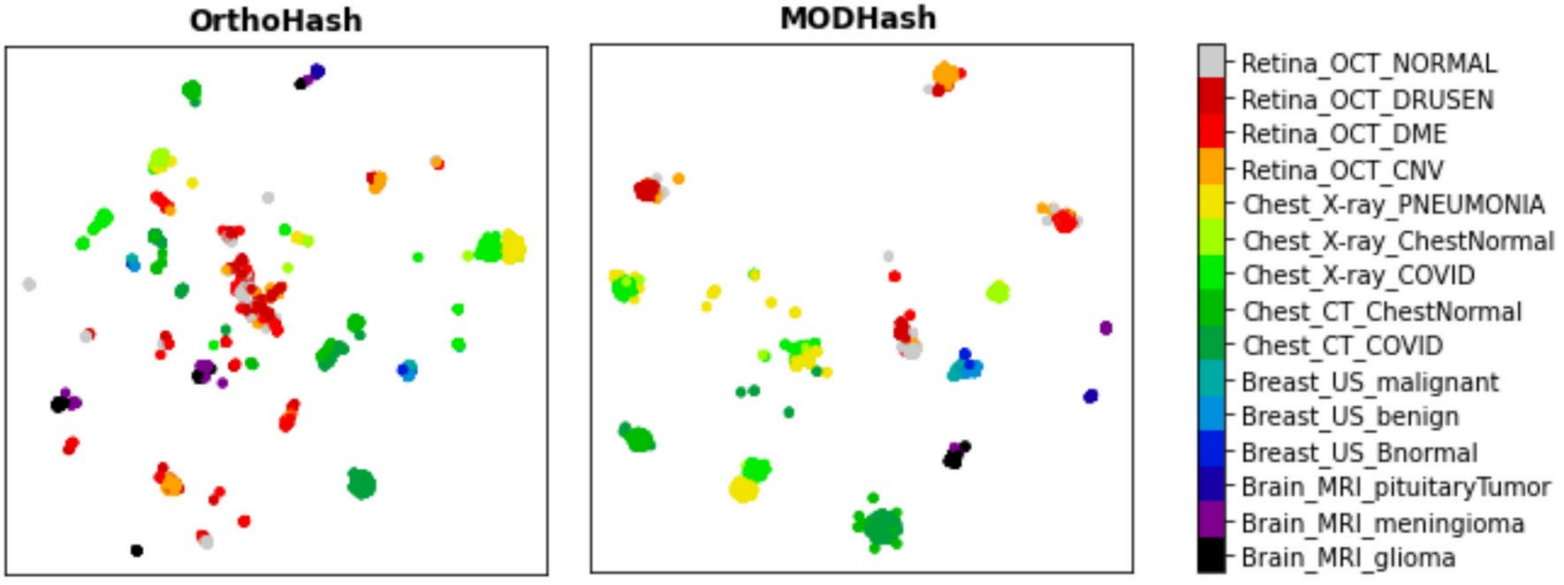
The UMAP visualizations for OrthoHash and MODHash.

OrthoHash achieves the best results among SOTA methods. In the scenarios with $\mathcal {M}$, $\mathcal {O}$, it is evident that OrthoHash and MODHash exhibit similar performance. However, in the remaining cases, particularly those with disease-related specifications, MODHash showcases significantly superior results. It achieves a substantial 12% increase in *mAP*@*p* and a 2% enhancement in *nDCG*@*p* compared to SOTA. UMAP (https://umap-learn.readthedocs.io/en/latest/) is utilized to visualize the projection of all possible different combinations of characteristics in the dataset. The visual comparison with OrthoHash of our results, as illustrated in Fig. 4. The projection of generated binary hash codes for similar images from MODHash indicates that these codes are closer together in the reduced-dimensional space compared to those generated by OrthoHash.

**Table 4:** mAP comparison between $MODHash-64$ and SOTA.

**Metric**	**Method**	$\mathcal {M}$	$\mathcal {O}$	$\mathcal {D}$	$\mathcal{M}\mathcal{O}$	$\mathcal{M}\mathcal{D}$	$\mathcal{O}\mathcal{D}$	$\mathcal {MOD}$
*mAP*@10	HashNet	0.6639	0.7730	0.4280	0.6640	0.3821	0.4280	0.3821
	DCH	0.9350	0.9383	0.5587	0.9350	0.5560	0.5587	0.5567
	OrthoHash	0.9944	0.9971	0.7548	0.9944	0.7501	0.7526	0.7501
	PCDH	0.9985	0.9989	0.7638	0.9985	0.7636	0.7638	0.7636
	HSCDA	0.7506	0.8503	0.5477	0.7506	0.4244	0.4477	0.4244
	HSDH	0.7087	0.8294	0.5094	0.7087	0.4285	0.5094	0.4285
	MODHash	**0.9992**	**0.9998**	**0.8341**	**0.9992**	**0.8336**	**0.8341**	**0.8336**
*mAP*@100	HashNet	0.5698	0.7098	0.3223	0.5698	0.2657	0.3223	0.2657
	DCH	0.9206	0.9240	0.4714	0.9206	0.4690	0.4714	0.4696
	OrthoHash	0.9925	0.9956	0.7127	0.9925	0.7099	0.7127	0.7099
	PCDH	0.9944	0.9947	0.7026	0.9944	0.7025	0.7026	0.7025
	HSCDA	0.6056	0.7665	0.3996	0.6056	0.3380	0.3996	0.3380
	HSDH	0.5924	0.7339	0.3814	0.5924	0.3312	0.3814	0.3312
	MODHash	**0.9939**	**0.9998**	**0.8041**	**0.9983**	**0.8036**	**0.8042**	**0.8037**
*mAP*@1000	HashNet	0.4950	0.6557	0.2675	0.4950	0.2050	0.2675	0.2050
	DCH	0.8960	0.9015	0.4292	0.8960	0.4268	0.4292	0.4271
	OrthoHash	0.9770	0.9805	0.6859	0.9770	0.6865	0.6859	0.6865
	PCDH	0.9170	0.9176	0.6535	0.9170	0.6547	0.6535	0.6547
	HSCDA	0.5650	0.7166	0.3564	0.5650	0.3027	0.3564	0.3027
	HSDH	0.5545	0.7192	0.3550	0.5645	0.3032	0.3550	0.3032
	MODHash	**0.9929**	**0.9998**	**0.7850**	**0.9979**	**0.7845**	**0.7850**	**0.7845**

Bold values represent the best performance score among all the methods.

**Table 5:** nDCG comparison between $MODHash-64$ and SOTA.

**Metric**	**Method**	$\mathcal {M}$	$\mathcal {O}$	$\mathcal {D}$	$\mathcal{M}\mathcal{O}$	$\mathcal{M}\mathcal{D}$	$\mathcal{O}\mathcal{D}$	$\mathcal {MOD}$
*nDCG*@10	HashNet	0.7399	0.8392	0.7061	0.8046	0.7093	0.7455	0.7406
	DCH	0.9352	0.9537	0.7625	0.9532	0.8432	0.8440	0.8656
	OrthoHash	0.9953	0.9977	0.8243	0.9974	0.9283	0.9286	0.9410
	PCDH	0.9990	0.9990	0.8852	0.9994	0.8986	0.9035	0.9484
	HSCDA	0.6149	0.6149	0.7564	0.6217	0.7547	0.7143	0.5211
	HSDH	0.6203	0.6203	0.6537	0.6804	0.6503	0.6415	0.5738
	MODHash	**0.9993**	**0.9999**	**0.8837**	**0.9999**	**0.9625**	**0.9625**	**0.9692**
*nDCG*@100	HashNet	0.8001	0.8627	0.6876	0.8315	0.7195	0.7542	0.7527
	DCH	0.9614	0.9602	0.7826	0.9597	0.8567	0.8572	0.8778
	OrthoHash	0.9956	0.9977	0.8632	0.9973	0.9390	0.9393	0.9508
	PCDH	0.9983	0.9983	0.8878	0.9984	0.9013	0.9056	0.9507
	HSCDA	0.6891	0.6891	0.6002	0.6923	0.5474	0.5731	0.6027
	HSDH	0.6961	0.6961	0.5933	0.7017	0.5553	0.5732	0.6014
	MODHash	**0.9994**	**0.9994**	**0.9033**	**0.9999**	**0.9639**	**0.9639**	**0.9713**
*nDCG*@1000	HashNet	0.8306	0.8876	0.7612	0.8644	0.7778	0.8061	0.8050
	DCH	0.9553	0.9639	0.8352	0.9634	0.8912	0.8918	0.9067
	OrthoHash	0.9935	0.9953	0.8977	0.9950	0.9536	0.9539	0.9625
	PCDH	0.9860	0.9860	0.9069	0.9861	0.9152	0.9180	0.9514
	HSCDA	0.7782	0.7782	0.6928	0.7883	0.6566	0.6770	0.6999
	HSDH	0.7784	0.7784	0.6929	0.7884	0.6563	0.6768	0.6991
	MODHash	**0.9994**	**0.9994**	**0.9294**	**0.9999**	**0.9717**	**0.9718**	**0.9777**

Bold values represent the best performance score among all the methods.

**Fig. 5:**
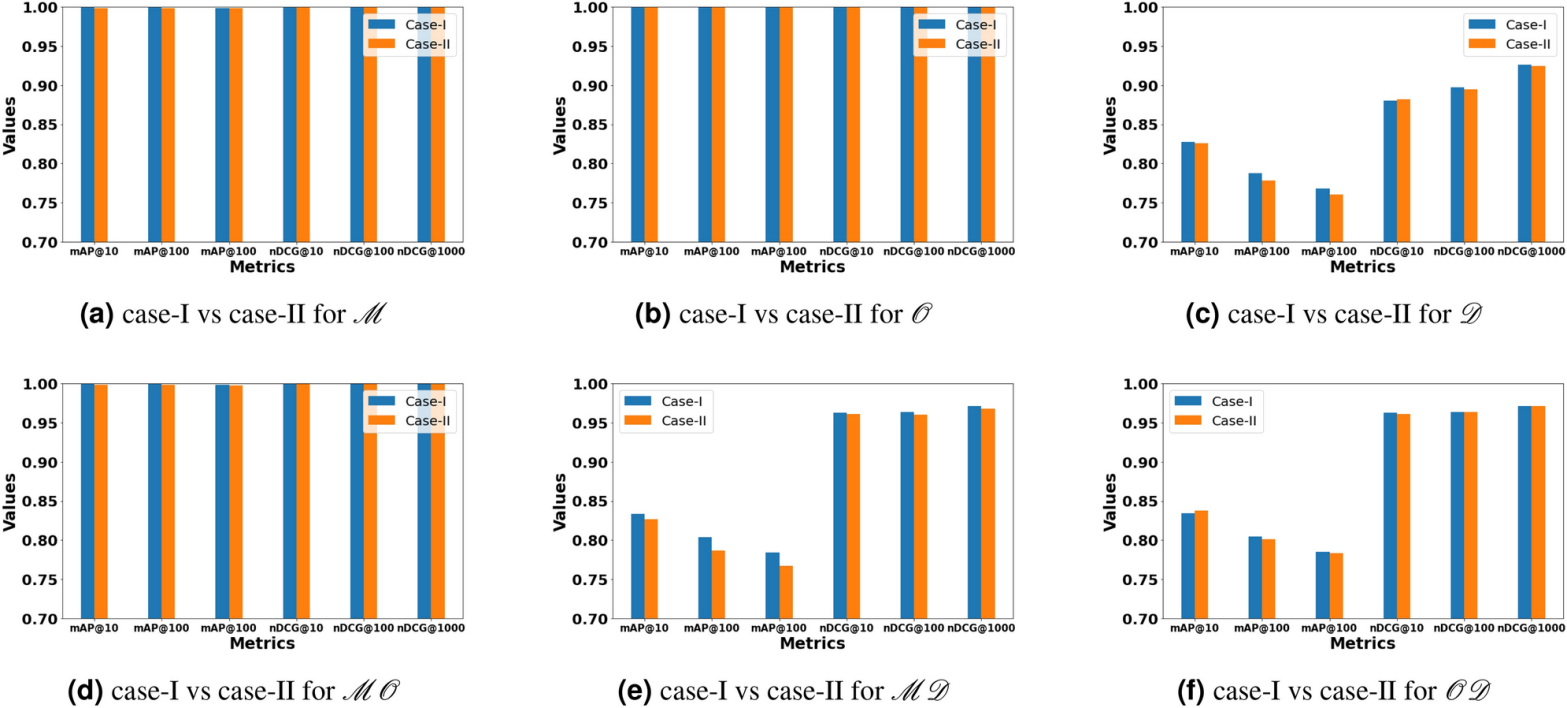
A visual comparison between case-I and case-II for $MODHash-64$.

### Full hash code VS characteristic-specific hash code

Previous experiments are evaluated based on the Hamming distance of full hash code length *K*, i.e., $d_H(\textbf{b}_r^G, \textbf{b}_q^Q)$. The effect of hash code generation on image characteristics is analyzed by measuring performance using Hamming distance in two distinct manners.

- **Case-I:** Compute the Hamming distance between the two final hash codes, i.e., $d_H(\textbf{b}_r^G, \textbf{b}_q^Q)$, and perform retrieval depending upon the specific case. This case is similar to previous experiments.
- **Case-II:** Compute the Hamming distance between two specific hash codes, i.e. $d_H(\textbf{b}_{1r}^G, \textbf{b}_{1q}^Q)$ or $d_H(\textbf{b}_{2r}^G, \textbf{b}_{2q}^Q)$ or $d_H(\textbf{b}_{3r}^G, \textbf{b}_{3q}^Q)$ depending upon the specific case.

The qualitative performance comparison of $MODHash-64$ between Case-I and Case-II is also provided in Fig. 5. It is observed that the performance levels between Case-I and Case-II are quite similar for $MODHash-64$. This suggests that full hash code and characteristic-specific hash code carry identical information for $MODHash-64$.

### Ablation study

Three different training protocols are chosen to observe the effect of the different loss functions on retrieval performance. Firstly, minimizing only $L_{1}$ focuses on differentiating classes of different characteristics and checking the retrieval performance. Table 6 shows that MODHash achieves nDCG@100 of 0.9669 and mAP@100 of 0.7938 after employing only classification losses. Subsequently, the retrieval performance is measured after adding the similarity loss ($L_2$) and quantization loss ($L_3$) with the existing classification loss ($L_1$). It is observed from Table 6 that after employing $L_2$ and $L_3$, results are gradually improved.

**Table 6:** Ablation study for $MODHash-64$ with baseline methods.

Employed losses	nDCG@10	nDCG@100	nDCG@1000	mAP@10	mAP@100	mAP@1000
$L_1$	0.9644	0.9669	0.9714	0.8325	0.7938	0.7476
$L_1 + L_2$	0.9671	0.9688	0.9763	**0.8368**	0.7956	0.7837
$L_1 +~L_2 + \alpha L_3$	**0.9692**	**0.9713**	**0.9777**	0.8336	**0.8037**	**0.7845**

Bold values represent the best performance score among all the methods.

## Conclusion

This work designs an effective structure-based CBMIR system termed MODHash by utilizing images of multiple modalities, organs, and diseases. MODHash is based on DNH, which learns to generate image-dependent hash codes from images by its structure property. It consists of characteristic classification losses to obtain distinct features for different characteristics, Cauchy entropy loss to decrease HD between most similar images, and quantization loss for bit regularization. The improved performance can be directly attributed to modified loss functions that jointly optimize the network binary representation learning. Comprehensive experiments are conducted on large datasets, and the retrieval performance is compared with SOTA. Our method outperforms SOTA in terms of both mAP and nDCG metrics. The experimental results have shown that the proposed approach is a promising solution for next-generation medical imaging retrieval systems.

## Data Availability

The utilized datasets in the current study are available in the Figshare, Mendeley, and Kaggle repositories, (Link1, Link2, Link3, Link4)
